# COVID-19 and *Clostridioides difficile* Coinfection Analysis in the Intensive Care Unit

**DOI:** 10.3390/antibiotics13040367

**Published:** 2024-04-17

**Authors:** Mircea Stoian, Adina Andone, Alina Boeriu, Sergio Rareș Bândilă, Daniela Dobru, Sergiu Ștefan Laszlo, Dragoș Corău, Emil Marian Arbănași, Eliza Russu, Adina Stoian

**Affiliations:** 1 Department of Anesthesiology and Intensive Care, George Emil Palade University of Medicine, Pharmacy, Sciences and Technology of Targu Mures, 540139 Targu Mures, Romania; mircea.stoian@umfst.ro; 2 Gastroenterology Department, George Emil Palade University of Medicine, Pharmacy, Sciences and Technology of Targu Mures, 540142 Targu Mures, Romania; aboeriu@gmail.com (A.B.); daniela.dobru@umfst.ro (D.D.); 3 Orthopedic Surgery and Traumatology Service, Marina Baixa Hospital, Av. Alcade En Jaume Botella Mayor, 03570 Villajoyosa, Spain; sergiob1976@gmail.com; 4 Intensive Care Unit, Mures, County Hospital, Street Gheorghe Marinescu No 1, 540136 Targu Mures, Romania; laszlo.sergiu.stefan@gmail.com (S.Ș.L.); dragos.corau@gmail.com (D.C.); 5 Department of Vascular Surgery, George Emil Palade University of Medicine, Pharmacy, Science and Technology of Targu Mures, 540139 Targu Mures, Romania; emilarbanasi1@gmail.com; 6 Clinic of Vascular Surgery, Mures County Emergency Hospital, 540136 Targu Mures, Romania; eliza.russu@umfst.ro; 7 Doctoral School of Medicine and Pharmacy, George Emil Palade University of Medicine, Pharmacy, Sciences and Technology of Targu Mures, 540142 Targu Mures, Romania; 8 Department of Pathophysiology, George Emil Palade University of Medicine, Pharmacy, Sciences and Technology of Targu Mures, 540136 Targu Mures, Romania; adina.stoian@umfst.ro

**Keywords:** *Clostridioides difficile*, SARS-CoV-2, antibiotic therapy, corticosteroid therapy, intensive care unit

## Abstract

Since the emergence of SARS-CoV-2 in late 2019, the global mortality attributable to COVID-19 has reached 6,972,152 deaths according to the World Health Organization (WHO). The association between coinfection with *Clostridioides difficile* (CDI) and SARS-CoV-2 has limited data in the literature. This retrospective study, conducted at Mureș County Clinical Hospital in Romania, involved 3002 ICU patients. Following stringent inclusion and exclusion criteria, 63 patients were enrolled, with a division into two subgroups—SARS-CoV-2 + CDI patients and CDI patients. Throughout their hospitalization, the patients were closely monitored. Analysis revealed no significant correlation between comorbidities and invasive mechanical ventilation (IMV) or non-invasive mechanical ventilation (NIMV). However, statistically significant associations were noted between renal and hepatic comorbidties (*p* = 0.009), death and CDI-SARS-CoV-2 coinfection (*p* = 0.09), flourochinolone treatment and CDI-SARS-CoV-2 infection (*p* = 0.03), and an association between diabetes mellitus and SARS-CoV-2-CDI infection (*p* = 0.04), as well as the need for invasive mechanical ventilation (*p* = 0.04). The patients with CDI treatment were significantly younger and received immuno-modulator or corticotherapy treatment, which was a risk factor for opportunistic agents. Antibiotic and PPI (proton pump inhibitor) treatment were significant risk factors for CDI coinfection, as well as for death, with PPI treatment in combination with antibiotic treatment being a more significant risk factor.

## 1. Introduction

The literature has limited data regarding the association between coinfection with *Clostridioides difficile* and SARS-CoV-2 in Intensive Care Units. Most of the publications have assessed the characteristics of *C. difficile* comparatively between the COVID-19 pandemic and the pre-pandemic period. Starting from this comparison, our goal was to analyze the data of critical patients admitted to the Intensive Care Unit (ICU) with SARS-CoV-2 infection and compare them with critical patients with CDI admitted to the ICU.

Since the end of 2019, global healthcare has been affected by the COVID-19 pandemic and its consequences. At the end of October 2023, nearly 4 years after the onset of the pandemic, 771,408,825 confirmed COVID-19 cases were declared, with a total number of 6,972,152 cumulative deaths. There are still reports of new-onset cases, without an epidemiological risk of a new pandemic [1]. In Romania, the first case of COVID-19 was identified on the 15 February 2020, and since then, more than 3,485,792 COVID-19 cases were reported, with a cumulative 68,455 death cases.

COVID-19 can present with various associated symptoms, varying from loss of smell to nasal obstruction, fever, or difficulty breathing. Interestingly, diarrhea was reported with an incidence of 19% [2]. Most of the COVID-19 cases had a mild or moderate severity and did not require hospitalization or advanced medical care [3]. Severe forms of COVID-19 manifested with pneumonia and difficulty breathing, with associated pulmonary infiltrations evidenced by thoracic imagistic scans. Pneumonia can be complicated by respiratory insufficiency, which requires oxygen supplementations or mechanical ventilation (invasive or non-invasive) [4,5]. Other severe complications of COVID-19 include thromboembolic events (pulmonary embolism or stroke), circulatory shock, myocardial lesions, arrhythmias and encephalopathies [6,7], loss of taste and smell [8], generalized headache [9], dizziness with vertigo [10], seizures [11], encephalitis [12], and Guillain–Barré syndrome [13]. Nevertheless, the exact mechanisms of these changes are still being studied. Several digestive symptoms, such as nausea, vomiting, diarrhea, and acute abdominal pain, were observed in COVID-19 cases [14].

The severe form of COVID-19 usually appears one week after the first symptoms, and the clinical manifestations can become catastrophic. Observational studies emphasized the role of dysbiosis in acute and post-acute COVID-19 and its connection to the severity of the disease [15]. The COVID-19 pandemic placed a significant burden on worldwide healthcare through the economic challenges related to increasing medical healthcare costs and the incidence of medical-system-associated infections (nosocomial infections). One of the most frequently associated infections is *C. difficile* infection (CDI). The incidence of CDI has been reported with an increasing pattern since the onset of the COVID-19 pandemic [16,17]. Multiple mechanisms have been proposed to explain the association between COVID-19 and CDI, from which we mention intestinal dysbiosis, the lack of adherence to proper hand hygiene, and excessive empiric antibiotic treatment [18,19,20]. The increasing use of antibiotics during the COVID-19 pandemic, especially for treating bacterial coinfections and as a prophylactic measure, could have contributed to a higher incidence of CDI and the development of resistant strains of *C. difficile.* Excessive use of antibiotics modifies the normal intestinal flora, which can lead to an increased risk of CDI [18]. CDI is easily transmitted through spores and appears in medical healthcare and the community [21]. The associated symptoms vary from mild diarrhea to toxic megacolon, which can become life-threatening.

While a part of the literature data available show an increased incidence of CDI during the COVID-19 pandemic, other studies show a weak association between the two types of infection [22]. Comparisons between the pre-COVID-19 incidence of CDI and the COVID-19 pandemic CDI infection levels showed a positive correlation between the two disorders, nevertheless showing uncertain significance [23].

This study aimed to assess whether the incidence of CDI and the severity of the clinical and microbiological forms of CDI are different between COVID-19 patients and non-COVID-19 patients during the pandemic.

It is essential to acknowledge the mechanism by which concomitant infection with CDI affects patients and their medical care.

## 2. Results

### 2.1. Hospital Characteristics

Mures County Clinical Hospital has 1182 beds, and it is the second hospital for number of beds in Romania, with a total of 44 beds assigned to the ICU (23 beds for the General (Polyvalent) Intensive Care Unit and 21 beds for the Postanesthesia Care Unit Intermediate Intensive Therapy (PACU). In February 2020, the hospital was assigned to the care of COVID-19 patients exclusively, as per the order of the Ministry of Health nr. 444; therefore, the admissions were open only for COVID-19 patients or other medical or surgical emergencies related to these patients. This led to a decrease of 50% in the number of admissions in comparison to previous years.

### 2.2. CDI Patient Characteristics

In the two years, between 30 March 2020 and 31 March 2022, a total of 19,414 patients were admitted in Mures County Clinical Hospital (HCM), from which 6340 patients were admitted with different forms of COVID-19 and 368 patients were diagnosed with CDI. In the ICU, without PACU, a total of 3002 critical patients were admitted: 1691 with SARS-CoV-2 infection; 1311 non-COVID-19 patients, of which 62 presented with CDI; and 38 had a coinfection with CDI and SARS-CoV-2.

**Figure 1:**
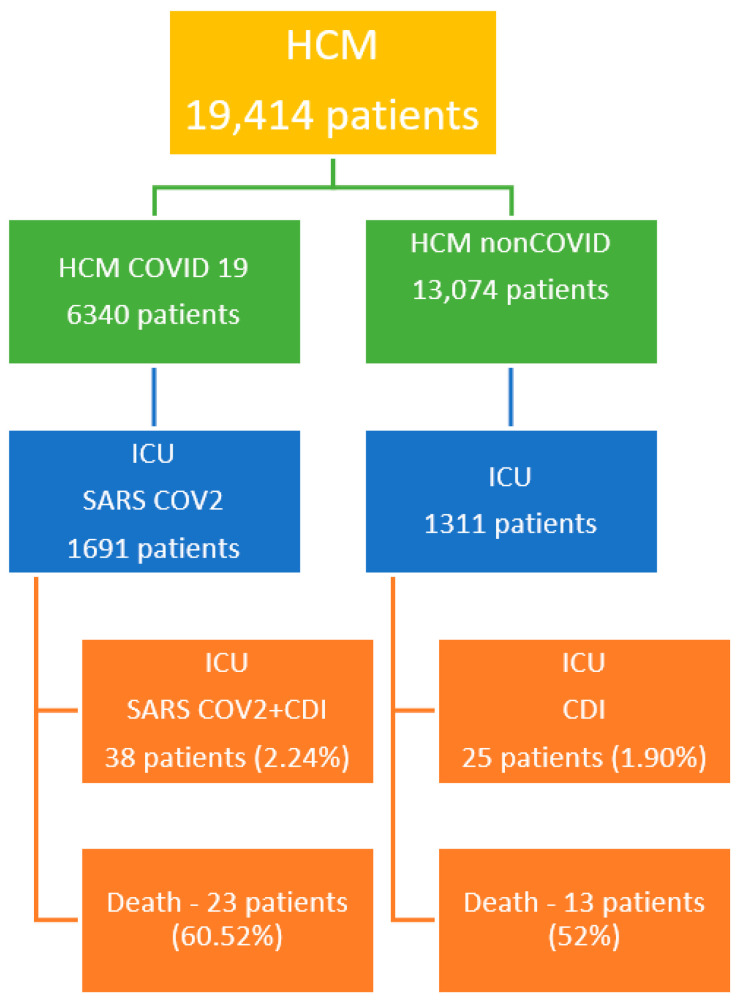
CDI patients’ characteristics.

All patients included are presented in Figure 1.

Thirty-six critical patients died during the ICU admission. Twenty-one patients from Group 1 came from an urban environment (55.26%).

The presence of a positive toxin A + B *C. difficile* infection was seen in 58 of the studied patients, and in 5 patients, we saw only toxin A *C. difficile*-positive tests.

**Figure 2:**
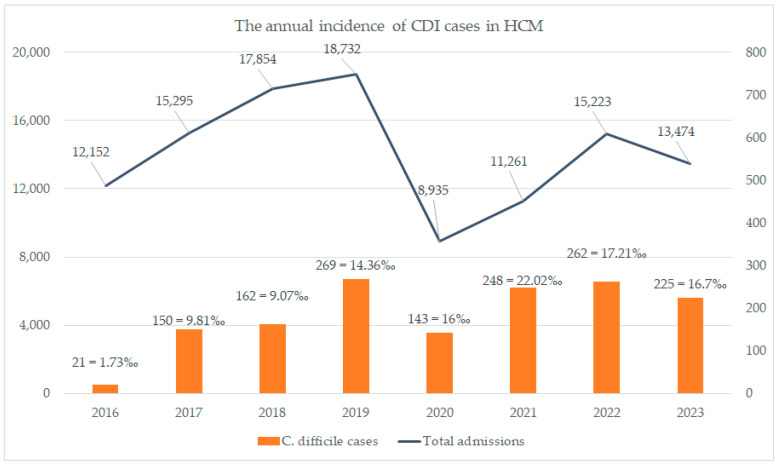
The annual incidence of CDI in HCM.

The annual incidence of CDI in Mures County Clinical Hospital is seen in Figure 2.

We can see in Figure 2 an annual increase in the CDI infections starting from 2020 (pandemic period) compared to the pre-pandemic period. In 2020 and 2021, our hospital admitted only COVID-19 cases to the ICU department at a proportion of 80%, showing a direct correlation of the increase in the incidence of CDI with COVID-19 cases, an incidence which dropped in the 2022 and 2023 periods, when our admission pool was mixed (COVID-19 and non-COVID-19 cases). In the remaining 20% of non-COVID-19 admissions, the percentage of CDI infection was very low (1.2 per 1000 cases).

### 2.3. Incidence Analysis

The total number of admissions is described in Figure 2. We see an increase in CDI incidence in the pre-pandemic period, which varies from 0.8 up to 5.29 per 1000 patients, which increased during the pandemic period, varying from 6.02 per 1000 patients, when we admitted only COVID-19 cases in the HCM. This represents the total number gathered from our electronic databases of nosocomial infections. The patients were not admitted with a diagnosis of CDI before their admission.

In the ICU department, we found 51 cases during 2020–2021 (COVID-19 group), with an incidence of 5.6/1000 cases discharged, and 99 cases during 2017–2018 (pre-COVID-19 group), with an incidence of 6.1/1000 cases discharged (*p* = 0.6). Although the incidence in our selected patients was not statistically different, the annual incidence as seen in Figure 2 has risen, taking into consideration there were only COVID-19 patients admitted in the ICU department.

We found 38 cases of CDI in the COVID-19 group, with an incidence reported for all admitted patients in the ICU of 2.24%, and 25 cases in the non-COVID-19 group, with an incidence of 1.83%. The mean age of the COVID-19 group was 68.79 ± 16.24 years, whereas in the non-COVID-19 group, it was 68.83 ± 12.83 years during the 2-year period of analysis.

The mean duration of ICU admissions in the COVID-19 group was 13.57 ± 8.34 days, whereas in the non-COVID-19 group it was 9.21 ± 9.02 days.

Comparing the mean results, we have a significant statistical difference between the two groups (CDI + COVID-19 group and CDI group) regarding Carmeli score (*p* < 0.01), leukocyte count both at admission and at discharge (*p* = 0.01, *p* = 0.04), neutrophil count at admission and at discharge (*p* = 0.03, *p* = 0.01), creatinine level (*p* = 0.001), ALT (*p* = 0.05), LDH (*p* = 0.025), and platelet count (*p* < 0.01). The paraclinical evolution of patients is seen in Table 1.

Regarding the antibiotic treatment administered, we had a number of patients on cephalosporin treatment (n = 21), carbapenems (n = 11), fluoroquinolones (n = 5), beta-lactamines (n = 6), and aminoglycosides (n = 5). All antibiotic treatments were administered before the hospital admission, there were no patients with previous antibiotic therapy, and not all patients with SARS-CoV-2 infection received antibiotic treatment.

Fluoroquinolone treatment was statistically associated with cephalosporine treatment (*p* = 0.03, Chi-Square test), beta-lactamine, and cephalosporine treatment (*p* = 0.001, Chi-Square test).

Death and CDI-SARS-CoV-2 coinfection were statistically significantly associated with PPI treatment (*p* = 0.01, Chi-Square test; *p* = 0.03, Chi-Square test).

From all patients included in this study who died during ICU admission, 75% of deaths occurred in CDI-SARS-CoV-2 coinfection patients (*p* = 0.09, Chi-Square test).

### 2.4. Comorbidities Analysis

We found no statistical differences by performing the analysis between the two groups regarding hepatic comorbities, diabetes mellitus, renal comorbidities, cardiovascular comorbidities, antibiotic treatment administered, and sex.

There was a statistically significant association between renal and hepatic comorbidities (*p* = 0.009, Chi-Square test).

CDI + SARS-CoV-2 coinfection was statistically associated with death (*p* = 0.01, Chi-Square test) as well as the status of ventilation (*p* = 0.02, Chi-Square test).

As far as comorbidities, the only comorbidity that was associated with death was diabetes mellitus (*p* = 0.05, Chi-Square test) in the CDI + SARS-CoV-2 group.

There was an association between the need for invasive ventilation and CDI + SARS-CoV-2 coinfection (*p* = 0.00, Chi-Square test), and diabetes mellitus was statistically associated with CDI + SARS-CoV-2 infection (*p* = 0.04, Chi-Square test) and the need for invasive ventilation (*p* = 0.04, Chi-Square test).

Gastrointestinal symptoms such as anorexia, nausea, vomiting, and abdominal pain were reported in 5 patients in the SARS-CoV-2 group (20%) and 16 patients in the SARS-CoV-2-CDI group (42.1%). There were no associated gastrointestinal disorders in either of the two groups studied.

### 2.5. Severity Score Analysis

The Carmeli score calculated at admission was 1.79 + 0.74 in the COVID-19/-CDI coinfection patients and 2.46 ± 0.5 in patients with CDI (*p* = 0.001, Student *t*-test). The APACHE score in CDI + COVID-19 coinfection patients was 18.08 ± 4.68 and 25 ± 8.54 in the CDI group (*p* = 0.06, Student *t*-test). The SOFA median score was 8.96 ± 2.05 in CDI + COVID-19 coinfection patients, and 9 ± 3.5 in CDI patients (*p* = 0.965, Student, *t*-test).

## 3. Discussion

The COVID-19 pandemic represented an unprecedented health crisis, bringing about radical changes in medical practice in a very short time. The implementation and strong adherence to infection prevention protocols, wearing protective equipment, and improving hand hygiene are considered changes that have influenced the decrease in the incidence of hospital-acquired infections, including CDI [24,25,26]. Analyzing the incidence of CDI from 2016 to 2023 at our hospital, we observe a progressive increase in the number of diagnosed cases of CDI rather than a decrease as expected. If in 2016 the number of cases was 1.73‰, it progressively increased to 9.81‰ in 2017, 14.36‰ in 2019, 16‰ in 2020, reaching the highest incidence of 22.02‰ in 2021, followed by a slight decline to 17.21‰ in 2022, and then 16.7‰. An increase in CDI incidence is reported in other studies, explaining that this rise is because of increased use of antibiotics and/or steroids, or even the modification of the patient population profile admitted during the pandemic [18,27,28,29]. In another study, Markovic-Denic L reported that the incidence density rate was three times higher when the hospital was a dedicated COVID-19 hospital, meaning only COVID-19-positive patients were admitted, compared to the period when it was a non-COVID-19 hospital, before and during the COVID-19 pandemic [30]. Kuijper EJ et al. show that CDI incidence increased in the mid-first decade of the 21st century due to highly virulent new strains of C. difficile, such as ribotype (RT) 027 [31]. Following these findings, measures have been introduced for rational antibiotic use, infection prevention, and control, factors considered essential in CDI prevention, thus becoming a major national priority in many states [32].

Analyzing the incidence of CDI from 30 March 2020 to 31 March 2021 in critically ill patients in the ICU, we found a higher incidence of CDI in the COVID-19 group at 2.24%, compared to the non-COVID-19 group, with an incidence of 1.83%. The average age of the COVID-19 group was 68.79 ± 16.24 years, while in the non-COVID-19 group it was 68.83 ± 12.83 years over a period of 2 years of analysis. Boeriu et al. show an average age of 69.56 ± 12.389 in the COVID-19 group and 64.84 ± 15.78 in the non-COVID-19 group, with a higher incidence of CDI at older age (*p* = 0.025) [33]. In our study group, there were no significant differences in the incidence regarding the age of patients with CDI. The average length of stay in the ICU for the COVID-19 group was 13.57 ± 8.34 days, while for the non-COVID-19 group it was 9.21 ± 9.02 days, indicating the severity of COVID-19-CDI coinfection. These data highlight the prolonged hospitalization period and increased mortality of patients with dual infection. Gavrielatou et al. show that the average length of stay in the ICU for patients with COVID-19-CDI coinfection was 12 days (range 1–59 days), and the mortality of this group of patients increased to 7/11 (63%) [34]. Buetti N et al., using prospectively collected multicenter data, showed that the risk of COVID-19 patients admitted to the ICU developing BSI (blood stream infection) was higher than that for patients without COVID-19 after seven days of ICU stay. Clinicians should be particularly attentive to late ICU-BSI in patients with COVID-19 [35]. The literature data have focused on the evolution of patients with CDI and COVID-19 coinfection. Negative outcomes are associated with prolonged hospitalization of COVID-19 and CDI patients, as well as CDI recurrence after hospital discharge [36]. The incidence of mortality during ICU hospitalization of patients with CDI-SARS-CoV-2 coinfection was 75% (*p* = 0.09, Chi-Square test). Allegreti et al. showed a significantly higher mortality incidence in patients with associated infections (80% in COVID-19 with CDI versus 12.2% in patients with only COVID-19, *p* < 0.0001) [37], while Sandhu A et al. showed that four out of nine patients with COVID-19 and CDI coinfection died during hospitalization [38]. Awan RU et al. found that in-hospital mortality was significantly higher among patients with COVID-19 with CDI compared to patients with COVID-19 without CDI (23% vs. 13.4%, OR: 1.3, 95% CI: 1.2–1.5, *p* = 0.01) [16]. Carmeli score evaluation showed a significant statistical difference between the two groups (*p* < 0.01), highlighting once again the role of repeated hospitalizations and antibiotic therapy in triggering CDI infection. Van Rossen TM et al. identified the only risk factors associated with CDI recurrence as older age, healthcare-associated CDI, previous hospitalization (<3 months), PPI initiated during/after CDI diagnosis, and CDI recurrence, while for severe forms of CDI, only older age was identified as a risk factor [39].

The most prescribed antibiotics for the patients included in this study were cephalosporins (33.33%), carbapenems (17.46%), beta-lactams (9.52%), fluoroquinolones (7.93%), and aminoglycosides (7.93%). The association of antibiotic treatment with fluoroquinolone–cephalosporin (*p* = 0.03, Chi-Square test) and beta-lactams–cephalosporin (*p* = 0.001, Chi-Square test) in patients with coinfection is associated with an unfavorable outcome for them.

The statistical analysis in our study showed the association between comorbities in our selected patients and the intense use of antibiotic treatment; together with PPI treatment, it was a high-risk factor for CDI infection, which led to a higher fatality, even though the patients had respiratory distress and lung failure.

We observed a high percentage of patients with COVID-19-CDI coinfection who received antibiotic treatment with one or more class of antibiotics. This is one of the most important result in our study, as antibiotic use should be limited in patients at risk of developing CDI, and treatment guidelines in COVID-19 were modified at a late stage after SARS-CoV-2 detection with caution rules regarding antibiotic treatment. Despotović A et al. show that the most frequently used antibiotics in COVID-19 patients were macrolides (32.4%), cephalosporins (29.6%), and fluoroquinolones (28.2%). A third of patients (34.5%) reported using more than one antibiotic [40]. We found no statistical differences between hepatic comorbidities, diabetes mellitus, renal comorbidities, cardiovascular comorbidities, antibiotic treatment administered, and the sex of patients between the two studied groups. Diabetes is frequently observed in hospitalized patients with COVID-19, with a reported prevalence between 7 and 30%. It can lead to chronic inflammation and an exaggerated immune response, as observed in COVID-19 infection [41]. Chronic liver disease is known as an independent risk factor for CDI infection, as well as for COVID-19 infection, due to frequent hospitalizations and reduced immunity [16,42]. It has also been noted that patients with concurrent COVID-19 and CDI have a higher prevalence of essential hypertension (HTN), coronary artery disease (CAD), and congestive heart failure (CHF) [43]. A meta-analysis showed that pre-existing coronary artery disease (CAD) is present in about one-tenth of hospitalized patients with COVID-19 [44].

In our analysis, the only comorbidity associated with the death of patients with COVID-19-CDI coinfection was diabetes mellitus (*p* = 0.05, Chi-Square test). Diabetics have a higher antibiotic consumption due to frequent infections, thus increasing their predisposition to CDI due to intestinal dysbiosis [41,45,46], thereby triggering increased morbidity and mortality when combined. This could indicate the possibility of a genetic background being involved in the intestinal dysbiosis, and a possible genetic susceptibility to develop autoimmune gastrointestinal disorders even after this episode of CDI or CDI and SARS-CoV-2 coinfection. Also, the association with fatality of COVID-19 + CDI coinfection in patients with diabetes mellitus was proven in our study, compared to other comorbidities. Even though it is well known that diabetes mellitus is an important risk factor for fatality in COVID-19 cases, the role of CDI infection in these selected patients is significant.

Ghosdal et al. related in their study the incidence and significance of gastrointestinal symptoms in COVID-19 infection and concluded that a percentage as high as 25% of patients had gastrointestinal symptoms as the only manifestation, in relation to the disease severity and having high clinical implications [47].

In our study, the percentage was higher in the SARS-CoV-2 and CDI group, even though we excluded from the analysis diarrhea as a gastrointestinal symptom, showing the clinical implication of gastrointestinal symptoms even before the diagnosis of CDI. Taking this into consideration, we could conclude that SARS-CoV-2 infection facilitates CDI, and CDI does not worsen the gastrointestinal symptoms associated with SARS-CoV-2.

Yibirin et al., in their systematic review of the literature, presented the adverse effects associated with PPI use, signaling the risk of acquiring CDI, alongside respiratory infections, kidney disease, gastrointestinal malignancies, liver disease, and fracture risk [48].

In our study, the relation to death and PPI use was demonstrated only in the CDI-SARS-CoV-2 group, showing in fact the risk of PPI and CDI acquirement, which associated a higher risk of complications and fatality with these associated conditions.

CDI + SARS-CoV-2 coinfection is an independent factor of severity and is statistically associated with the death of patients (*p* = 0.01, Chi-Square test). The association of mechanical ventilation in patients with coinfection is associated with increased morbidity and mortality (*p* = 0.02, Chi-Square test).

Micek S.T et al. reported a mortality rate of 25.1% in patients with CDI requiring mechanical ventilation [44]

Through the analysis of the Carmeli score, calculated upon admission to the ICU for both groups, we obtained a value of 1.79 + 0.74 for COVID-19-CDI coinfection and 2.46 ± 0.5 in patients with CDI alone (*p* = 0.001, Student’s *t*-test). This score is used in Romania for screening patients susceptible to colonization with multidrug-resistant bacteria. In this study, the score emphasizes the risk of developing *C. difficile* infection, in addition to the risk of colonization with MDR germs.

The APACHE score in the group with CDI + COVID-19 coinfection was 18.08 ± 4.68 and, respectively 25 ± 8.54 in the CDI group (*p* = 0.06, Student’s *t*-test). Even in this severity score, calculated upon admission to the ICU, patients in the group with CDI infection without COVID-19 presented a higher risk of mortality. APACHE II is a predictive instrument that assesses the extent of a patient’s illness and predicts the prognosis of the disease, usually in terms of mortality, for patients admitted to the ICU [49]. Thus, we observe that although the group has an unfavorable prognosis, it has a better survival rate in the case of *C. difficile* mono-infection.

Another score evaluated upon admission to the ICU was the SOFA score, which had a value of 8.96 ± 2.05 in patients with concomitant infection with CDI + COVID-19 and 9 ± 3.5 in patients with CDI (*p* = 0.965, Student’s *t*-test). The differences between the two groups are practically statistically insignificant, so its use does not bring additional data for patients with COVID-19 infection. This was also observed by Moisa et al. in their study, where they proposed a new mortality predictability score at 28 days for patients admitted to the ICU [50].

Stoian et al. showed in a case presentation and literature review the significant number of patients which require ICU services due to severe respiratory, thrombotic, and septic complications and who require long-term hospitalization, by presenting a case of a 54-year-old woman with severe COVID-19 infection and an associated critical illness: polyneuropathy [51]. Although in our study we did not have cases with the critical illness polyneuropathy, the likelihood of them developing this condition was increased due to the association of both COVID-19 and *C. difficile* infection. The case presented was treated with high-dose intravenous immunoglobulins, which needs to be taken in to consideration if this pathology is suspected.

In other study by Stoian et al., the significance and large spectrum of the disease COVID-19 is presented by the study of the occurrence of acute disseminated encephalomyelitis in SARS-CoV-2 infection/vaccination [52]. We did not have cases of encephalomyelitis, but the rate of SARS-CoV-2 vaccination in our studied patients was extremely low, and neurological complications may have appeared in the long course of the disease, which was limited due to the *C. difficile*-associated infection.

In our previous study, published by Stoian et al., we followed up patients in the ICU, with a focus on long-term radiological pulmonary changes in mechanically ventilated patients with respiratory failure due to SARS-CoV-2 infection [5].

The radiological pulmonary changes were also recorded in our patients, but we did not follow them up radiologically, due to the increased mortality rate due to the association of these two separate conditions: SARS-CoV-2 infection and *C. difficile* infection.

In their study regarding the evolution and incidence of COVID-19 and CDI in a Romanian institution, Marinescu et al. showed that CDI has complicated the outcome of COVID-19 patients, especially for those with comorbidities or previously exposed to the healthcare system, and addressed the same need for vigilance for the extensive use of antibiotics, similar to our study [53].

### Study Limitations

Since only critically ill patients are admitted to the ICU, this study included a limited number of patients, leading to a small sample size. Additionally, this study was conducted in a single medical unit and was not a multicenter study. The number of patients admitted during that period to our unit was limited to medical emergencies and COVID-19-positive patients.

The evaluation of severity scores such as APACHE II and SOFA is limited to patients with COVID-19-CDI coinfection; thus, more comprehensive studies or meta-analyses of these predictability scores are needed.

## 4. Materials and Methods

### 4.1. Data Collection and Analysis

We performed a retrospective observational study using the data offered by the informatic system of Mures County Clinical Hospital and the analysis of the observational charts and the surveillance charts of CDI patients with COVID-19 infection admitted to the ICU. The follow-up period was 2 years, from the moment of admission of the first COVID-19 patient in the ICU on 31 March 2020 up until 31 March 2022.

We collected and analyzed the following data: age, environment, sex, comorbidities, incidence of CDI in the pre-COVID19 period and COVID-19 period, APACHE score, SOFA score, duration of treatment in the ICU, the status of mechanical ventilation, treatment administered (H2 blockers, PPI, empirical antibiotic treatment, the class of antibiotics administered), the identified pathogen, and documented infections. The following paraclinical examinations were followed up: leukocyte count, neutrophil count, lymphocyte count, monocyte count, platelet count, seric urea, creatinine, ALT, AST, GGT, LDH, ferritin, albumin, total proteins, glycemia, potassium, sodium, detection of toxin A + B *C. difficile*, and RT PCR SARS-CoV-2 status.

All the paraclinical examinations were conducted in the Mures County Clinical Hospital laboratory through spectrophotometry, flow cytometry, impedance testing, turbidimetry (PCR), molecular diagnosis, and immunochromatography for toxin A + B *C. difficile.*

We created 2 subgroups of patients: the first group—patients with SARS-CoV-2 and CDI coinfection—and the second group—patients with mono-infection with CDI.

Inclusion criteria:

- Critical patients admitted to the ICU department;
- SARS-CoV-2 infection, confirmed by RT-PCR SARS-CoV-2;
- Diagnosis of CDI, confirmed by toxin A + B *C. difficile*.

Statistical analysis was performed using software applications such as IBM SPSS Statistics v26 and Microsoft Excel 2019. We assessed parametric variables (ANOVA test), describing the data as continuous (mean, standard deviation [SD], median, min/max) depending on their distribution. We used correlations for quantitative variables with Pearson’s correlation coefficient (rho), with alpha set at 0.05. A *p*-value ≤ 0.05 was considered significant. Contingency tables and the Chi-Squared test were used to assess the correlation between the distributions of the categorical variables.

We analyzed two types of data: the incidence of CDI for Mures County Clinical Hospital in the 2016–2023 period and the comparative analysis of the two groups, coinfection with SARS-CoV-2 and CDI group and CDI group, during the two-year period.

### 4.2. Ethics Statement

For this study, we used data from the hospital’s informatic system and the observation charts and surveillance charts during the admissions. We did not collect supplementary data or materials or require any supplementary data from the patients included in this study.

The data do not include data regarding personal identity. We obtained the Ethics Committee of Mures County Clinical Hospital’s approval; 6077 from 7 April 2023.

## 5. Conclusions

The incidence of CDI infection in both COVID-19 and non-COVID-19 groups is practically similar. All patients underwent curative antibiotic treatment for a documented infection with at least one pathogen agent. The patients with CDI treatment were significantly younger and received immunomodulator or corticotherapy treatment, which was a risk factor for opportunistic agents.

Antibiotic and PPI treatment were significant risk factors for CDI coinfection, as well as for death, with PPI treatment associated with antibiotic treatment being a more significant risk factor. This was one of the most important aspects of our study, as COVID-19 treatment guidelines included, for a long period of time, recommendations for antibiotic treatment. Diabetes mellitus was a significant comorbidity associated with CDI-COVID-19 coinfections as well as death.

Most patients received empiric antibiotic treatment from the initial days of hospitalization.

For all patients admitted in the intensive care unit in a critical condition, the association of CDI with SARS-CoV-2 infection was associated with a worse prognosis and an increased risk of fatality.

## Figures and Tables

**Table 1:** Paraclinical examinations performed on admission and on discharge.

	Group	Admission	Discharge	Unpaired *t*-Test *p*
CRP (mg/dL)	1	36.32 ± 49.6	10.50 ± 11.80	0.731
	2	41.83 ± 72.76	12.95 ± 15.90	0.507
Fibrinogen (mg/dL)	1	475.88 ± 214.02	426.06 ± 187.94	0.374
	2	428.28 ± 184.23	439.59 ± 160.63	0.773
Ferritin (ng/mL)	1	1332.99 ± 901.43	2008.032 ± 2320.15	0.139
	2	838.05 ± 1176.75	724.33 ± 1120.29	0.07
LDH (U/L)	1	480.49 ± 231.26	510.43 ± 342.53	**0.02**
	2	319.00 ± 289.69	249.30 ± 101.82	**0.002**
Leucocytes (/mm^3^)	1	10.25 ± 5.06	16.51 ± 8.48	**0.01**
	2	13.67 ± 5.83	12.40 ± 5.66	**0.04**
AST (U/L)	1	57.89 ± 62.36	104.73 ± 268.58	0.08
	2	33.88 ± 28.20	34.46 ± 30.62	0.20
ALT (U/L)	1	74.43 ± 90.98	128.70 ± 235.63	**0.05**
	2	35.83 ± 30.47	32.17 ± 25.25	**0.05**
Creatinine (mg/dL)	1	1.07 ± 0.58	1.34 ± 1.28	**0.001**
	2	2.65 ± 2.73	1.95 ± 2.18	0.17
Urea (mg/dL)	1	70.52 ± 37.85	111.38 ± 93.41	0.06
	2	94.15 ± 60.59	83.90 ± 59.43	0.20

Group 1—COVID-19 + CDI; Group 2—CDI.

## Data Availability

Data are available based on request from the corresponding author.

## Abbreviations

ALT	alanine aminotransferase
APACHE II	Acute Physiology and Chronic Health Evaluation II
AST	aspartate aminotransferase
CAD	coronary artery disease
CDI	Clostridioides Difficile Infection
CHF	cardiac heart failure
GGT	gamma-glutamyl transferase
HCM	Hospital County Mures
HTN	hypertension
GDH	glutamate dehydrogenase
ICU	intensive care units
LDH	lactate dehydrogenase
MDR	multidrug resistance
RT PCR SARS-CoV-2	Real-Time Polymerase Chain Reaction SARS SARS-CoV-2
PPI	proton-pump inhibitors
PACU	Post-Anesthesia Care Unit
PCR	polymerase chain reaction
PCT	procalcitonin
SOFA	Sequential Organ Failure Assessment
VSH (ESR)	Erythrocyte Sedimentation Rate
